# Recent advances in cardiovascular disease research driven by metabolomics technologies in the context of systems biology

**DOI:** 10.1038/s44324-024-00028-z

**Published:** 2024-09-23

**Authors:** Boyao Zhang, Thierry Schmidlin

**Affiliations:** Institute of Immunology, University Medical Center of the Johannes Gutenberg-University Mainz, Mainz, Germany

**Keywords:** Systems biology, Metabolic disorders, Metabolism, Metabolomics

## Abstract

Traditional risk factors and biomarkers of cardiovascular diseases (CVD) have been mainly discovered through clinical observations. Nevertheless, there is still a gap in knowledge in more sophisticated CVD risk factor stratification and more reliable treatment outcome prediction, highlighting the need for a more comprehensive understanding of disease mechanisms at the molecular level. This need has been addressed by integrating information derived from multiomics studies, which provides systematic insights into the different layers of the central dogma in molecular biology. With the advancement of technologies such as NMR and UPLC-MS, metabolomics have become a powerhouse in pharmaceutical and clinical research for high-throughput, robust, quantitative characterisation of metabolic profiles in various types of biospecimens. In this review, we highlight the versatile value of metabolomics spanning from targeted and untargeted identification of novel biomarkers and biochemical pathways, to tracing drug pharmacokinetics and drug-drug interactions for more personalised medication in CVD research (Fig. 1).

## Introduction

By definition, cardiovascular disease (CVD) refers to diseases that are related to heart muscles and the vascular system supporting it. Ischemic heart disease (IHD), stroke and congestive heart failure (CHF) are the predominant types of CVD. In the early 1900s, CVD had been a leading cause of death in the developed world, accounting for 50% of deaths in high-income countries and 28% of deaths in low- and mid-income countries^1^. Despite the decline in age-adjusted death rates since 1950s^2^, according to the Global Burden of Diseases, Injuries, and Risk Factors Study (GBD) in 2019, among 369 diseases, CVD was still one of the leading causes of reduced health and life expectancy in people older than 50^3^. Therefore, there is an imminent need for more effective CVD management to improve people’s life quality and to relieve the burden CVD imposes on the healthcare system^4–7^. To this end, knowledge derived from conventional clinical monitoring or observations of the various types of risk factors (e.g. diet, medication, other diseases or disorders) and data-driven systematic characterisation of the genome, proteome, lipidome and metabolome will be useful for building a comprehensive knowledge base for biomarker identification and personalised disease prevention and treatment prognosis prediction^8–10^. In this review, we assess the values and limitations of the traditional CVD risk factors and discuss how omics technologies have facilitated the identification of new risk factors or biomarkers. We then focus on the added value of metabolomics approaches to CVD research by first highlighting its technological maturation from a qualitative description tool to a more quantitative molecular characterisation workhorse. We discuss the particular suitableness of metabolomics approaches for CVD biomarker identification by explaining the fundamental causes of CVD. Lastly, we provide an outlook for the application of metabolomics in areas that have not yet been the focus of CVD research, including host-microbiome interactions, drug-drug interactions and xenobiotic metabolism, which could be of great value for the discovery of new drug targets and personalised medication.

**Fig. 1:**
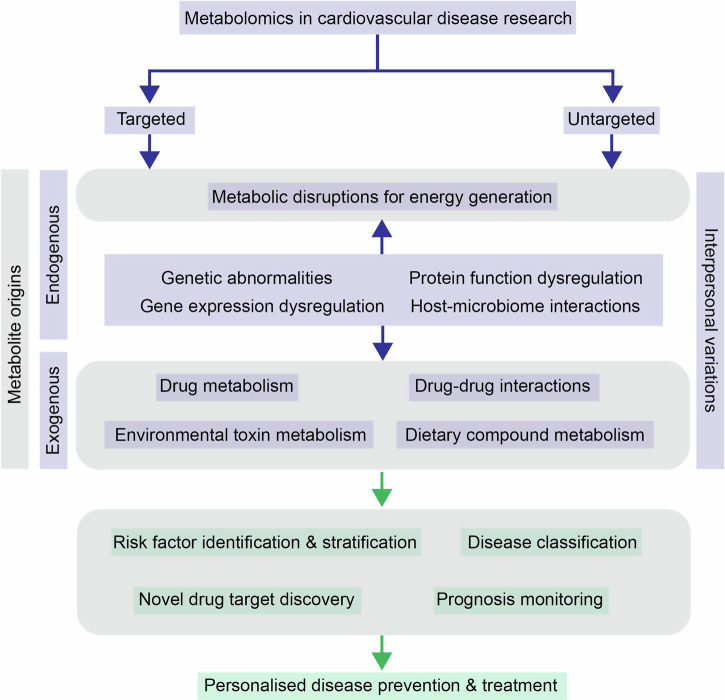
Summary of molecular mechanisms that can lead to cardiovascular diseases (CVD) development and how application of targeted and untargeted metabolomics approaches can help achieve more effective and personalised treatment and prevention by providing a more thorough understanding in various aspects.

## CVD risk factors derived from conventional clinical research

The top-down approach to prevent CVD could be to target the known risk factors based on epidemiological and clinical studies. As reviewed by American Society for Preventive Cardiology^11^, major risk factors for CVD are lifestyle-based ones such as unhealthy nutrition^12–15^, lack of physical activities^16–18^ and smoking^19,20^. The same risk factors also contribute to the development of disorders such as high blood pressure, overweight and obesity, diabetes and thrombosis, which are usually concomitant with CVD^11^. Adopting healthier lifestyles and habits is therefore generally advised under clinical settings to prevent multiple disorders that are interlinked with CVD. On the other hand, CVD risk can be lowered by targeting risk factors related to its concomitant diseases. For example, apolipoprotein B, non-high-density lipoprotein cholesterol (non-HDL-C) and low-density lipoprotein (LDL) particle number, which are parameters that are often at abnormal levels in dislipidemia, were found to be strong predictors of atherosclerotic CVD risk^21–23^ and this led to successful reduction in CVD risk by lowering these parameters with lipid management therapy^24–29^. Diabetes mellitus is yet another major risk factor for CVD, and treatments targeting diabetic biomarkers such as haemoglobin A1c, glucose, insulin and blood pressure are also known to be beneficial for CVD outcomes^30,31^. Additionally, ceramide lipids are predictive of both diabetes mellitus type 2^32^ and CVD^33–37^ development, which led to the implementation of CERT1 and CERT2 scores^38^. Chronic kidney disease (CKD) is considered to be another independent risk factor for atherosclerotic CVD due to its effects on endothelial dysfunction, atherosclerosis progression and hypertension. Hence biomarkers such as estimated glomerular filtration rate (eGFR) and urine protein excretion (i.e. albuminuria) are of predictive value for CVD where the level of eGFR is inversely correlated with CVD risk^39–41^. The advancements in CVD management by uncovering an increasing number of risk factors and their associated physiological mechanisms had led to some success in controlling the burden of the disease - age-standardised mortality rates (ASMRs) for CVD had declined by up to 47% in 15 European countries between 1900-2021^42^. However, as for many of the known diseases of multifactorial nature, there is no ‘one diagnosis or treatment for all’ for CVD and the burden it has on the healthcare systems is not yet relieved by managing the existing commonly known risk factors alone. Some of the key reasons for ineffective CVD management reside in the interindividual variations in terms of disease development and in treatment response^43–45^ as well as risk factors that are yet to be discovered and/or more accurately characterised^46^.

## CVD research in the omics era

As pointed out above, one of the major challenges in CVD prevention and treatment is the interindividual differences in disease progression and treatment responses, which is the result of various factors that are difficult to disentangle. For example, different people are exposed to different xenobiotics (e.g. dietary compounds, cigarette smoke) and hence different mixtures of exogenous risk factors. Even when exposed to the same xenobiotics, due to intrinsic differences (e.g. gender, family history, ethnicity), the level of risk exposure and actual risk imposed on each individual will be different, and qualitative characterisation of risk factors alone will not be sufficient to provide effective clinical solutions for individuals. To tackle such challenges, a solid understanding of CVD mechanisms and identification of more quantifiable and actionable clinical biomarkers (rather than descriptive or qualitative risk factors) are needed. This requires systematic characterisation of various types of biological molecules (e.g. DNA, RNA, proteins, lipids, metabolites) and analysis of the information flowing across different molecular layers (e.g. transcription, translation, enzymatic reactions) with laboratory-based technologies. Furthermore, to identify predictive/diagnostic/prognostic biomarkers that are clinically informative and actionable, sufficient statistical power is required when drawing conclusions, which requires large sample sizes. These requirements are fulfilled by the omics technologies which uncover an unprecedented amount of molecular information and have increasingly higher throughput and robustness. In the following section, we will focus on the deployments of omics technologies in CVD disease mechanism research and biomarker identification. Common terminologies used are explained in Box 1.

One potential molecular explanation for interindividual differences in CVD risk are variations in genetic makeup^47–49^. As genomes provide the blueprints for genetic products that have a diverse range of functions, it is not surprising that differences at the genomic level result in differences in the phenotypic disease development. Therefore, capturing genetic polymorphism in different individuals provides insights into the primary layer of molecular mechanisms of CVD. The ever advancing next-generation-sequencing (NGS) technologies are the powerhouse for genomic and genetic analyses. A plethora of genome-wide association studies (GWAS)^50^ had been conducted to uncover genotype-phenotype associations^51,52^. For example, locus 9p21 in the non-coding region initially elucidated in the Framingham Heart Study 100 K GWAS project^53^ had been reproducibly associated with coronary artery disease (CAD) and myocardial infarction across different populations^54–56^. With increasing size of cohorts in more diverse populations and large amounts of data aggregation, genotyping will undoubtedly uncover more genetic variants associated with CVD, which will provide a more comprehensive Mendelian paradigm of inherited heart diseases such as hypertrophic cardiomyopathy (HCM), dilated cardiomyopathy (DCM), arrhythmogenic cardiomyopathies and left ventricular non-compaction (LVNC)^57–59^. Family history and genetic testing have nowadays been incorporated into clinical settings to augment the diagnosis and classification of CVD^60,61^. For example, if a causative variant is identified in an individual diagnose with an inherited heart disease, other asymptomatic family of that individual can be offered to test for the causative variant too in the so-called proband genetic testing^60^.

Genomic analysis have already revealed a great deal of information about the possible involvement of different genes in CVD risk. However, the specific functions of different genetic products and the functional consequences of their interactions can only be revealed using techniques capturing the downstream biological processes such as transcription, translation, post-translational modifications and enzymatic activities. Transcription is the first layer of functional readout of DNA and the study of variations in splicing, levels of gene expression and regulation of non-coding RNA is referred to as transcriptomics^62,63^. Translation of RNA forms the next layer of functional readout, which encompasses protein abundances, post-translational modifications and protein-protein interactions, and is usually characterised by mass-spectrometry (MS)-based proteomics technologies or proximity extension assays^64^ (e.g. OLINK) where a detectable DNA amplicon is generated only when the coupled antibody binds to its antigen target. Lastly, as will be discussed in more detail in later sections, the closest phenotypic representation of the physiological state at a given time point, which integrates various endogenous and exogenous signals at the molecular level, is probably the metabolic profile within cells and tissues, which is characterised with metabolomics technologies. In the rest of this section, we provide examples of successful usage of multiomics technologies in biomarker identification and confirmation and mechanistic investigation in CVD research. We will also discuss the benefits of integrating data obtained with different omics approaches and the current challenges for multiomics data integration.

Due to its close vicinity to phenotypic manifestations and its ready clinical implementations, profiling of metabolites and lipids has successfully confirmed many of the classic risk factors discovered via traditional clinical observations. For example, branched-chain amino acids (BCAAs), which had been identified as biomarkers for various metabolic disorders, were confirmed to be associated with heart failure (HF) onset in prospective studies with quantitative measurements of BCAA levels in serum^65,66^. Additionally, by applying systematic profiling of lipidome in a prospective population-based study, novel panels of lipid markers for CVD were uncovered, providing more refined risk stratification^67^. At the protein level, proteomic analysis on 1129 proteins after planned myocardial injury also confirmed the significant and marked fold-changes in the clinically well-established biomarkers, troponin I and creatine kinase^68^. At the RNA level, novel roles of non-protein-coding RNAs including long non-coding RNAs (lncRNAs), microRNAs (miRNAs) and circular RNAs (cirRNAs) have been uncovered with the increasingly powerful next-generation sequencing (NGS) technologies, which mainly revolves around regulating normal functions in cardiomyocytes such as proliferation, differentiation, apoptosis, calcium homeostasis. Whilst still a relatively under-explored area, plenty of studies have already shown the association between circulating mRNA levels with CVD development and their potential roles of being clinical biomarkers^69–71^.

Indeed, by using large amounts of data obtained from cohort studies, associating signals at individual biological layers obtained with respective omics technologies with CVD risk already provides valuable insights into clinically relevant biomarkers. However, there is a constant flow of information across different biological layers, and information obtained from a single layer alone often leads to noise signals that mask the more biologically relevant ones, which inevitably reduces the predictive value of some of the biomarkers. Therefore, integrating signals from multiple biological levels is particularly useful in providing complementary information for filtration of the most relevant biochemical pathways that are potentially also more clinically actionable. For example, by combining genomics and transcriptomics, single nucleotide polymorphisms (SNPs) in the genome can be compared with gene expression profiles for the identification of expression quantitative trait loci (eQTLs), which regulate gene expression at *cis* or *trans* locations. The SNPs regulating *PCSK9* expression in abdominal fat were uncovered this way^72^ and due to their association with the plasma biomarker for CVD risks, LDL-C, such findings can potentially provide tissue-specific biomarkers or therapeutic targets for more accurate diagnosis and more effective treatment. Another example is that by correlating gene expression of key mediators of immune response with lipid levels, a potential mechanism of atherosclerosis, which is via lipid activation of immune cells, was elucidated^73^. Furthermore, with the increasing realisation of the role of gut microbiota in human health and disease regulation, genomic sequencing technologies have been more and more extensively used to characterise the diversity and relative abundances of microbial species in the gut, and cohort studies with metabolic profiling have shed light on the roles of microbial metabolites such as short-chain fatty acids (SCFAs) on CVD development via modulating inflammatory response^74,75^.

Another motivation for integrating information obtained from a diverse range of omics approaches is that each of them has their own methodological challenges that can result in reduced confidence in the conclusions drawn. For example, despite the fruitful outcomes derived from GWAS such as novel mechanism elucidation and more subtle disease classification, and advancement in data management strategies^76,77^, false associations due to population stratification still exist and the coverage of rare variants is incomprehensive^50,78^. As for transcriptomics and proteomics approaches, technical variations during sample preparation still pose a challenge for reliable and reproducible profiling (e.g. RNA is inherently unstable and its integrity might be affected during extraction, while the accuracy of protein quantification by proteomics might be affected by the extraction protocol and/or the chromatographic separation). On the other hand, whilst having more straightforward sample preparation procedure compared to genomics and transcriptomics and relatively comprehensive coverage of biologically relevant metabolites^79,80^, metabolic profiling also suffer from signal losses during sample preparation and instrumental measurements, and to explain the temporal changes in metabolic profiles with high confidence, sequential events happening at transcriptional, translational and protein modification levels need to be revealed. Hence, the reliability and accuracy of molecular pathways underlying the disease can potentially be enhanced by combining information uncovered via individual omics approaches^81,82^. On the other hand, despite the potential benefits of omics integration approaches, there are still challenges in such approaches due to the heterogeneous and complex nature of different types of data. Machine learning has received increasing attention to address such challenges as reviewed by Li et al.^83^.

Lastly, large cohort studies using human-derived samples are tempting for disease mechanism research given their direct organismic relevance and increased statistical power. However, in practical terms, sample types for such large-scale studies are often limited to peripheral tissues such as plasma and urine, which is beneficial for the overall sampling and data acquisition throughput and useful for correlational studies but can hinder the elucidation of organ-specific pathways. Tissue biopsies would be ideal for more localised mechanistic studies, but this introduces more time-consuming, invasive sampling procedures that are usually not deployed until more severe symptoms appear. One way to circumvent such lack of organ specificity is to use clinically relevant animal models, where the potential confounding factors (e.g. diet, genetics, age, gender) are controlled and samples can be taken from more diverse types of tissues. However, interspecies differences and the translational values of such models will need to be taken into consideration in such models as the interactions between various CVD risk factors are largely omitted since they are tightly controlled under experimental conditions, resulting in potentially misleading conclusions.

Box 1 *Risk factor*: a factor that increases the chance of developing a disease. This can be descriptive (e.g. age, family history, concomitant diseases, life habits) or measurable (e.g. blood pressure, serum cholesterol).

*Biomarker*: a quantifiable characteristic that indicates biological processes (e.g. disease) or response (e.g. medication).

*Omics technologies*: high-throughput technologies that comprehensively characterise a given biological system at the molecular level, including but not limited to genomics (analysis of complete sets of DNA), transcriptomics (analysis of complete sets of RNA transcripts), proteomics (analysis of protein structures, functions and activities) and metabolomics (analysis of small molecules - mainly endogenous metabolites and lipids, occasionally exogenous substances).

*Multiomics*: integrative analysis of results obtained from multiple omics technologies of the same sample or sample set.

## Mechanisms of CVD and versatile roles of metabolomics approaches in translational research

**Fig. 2:**
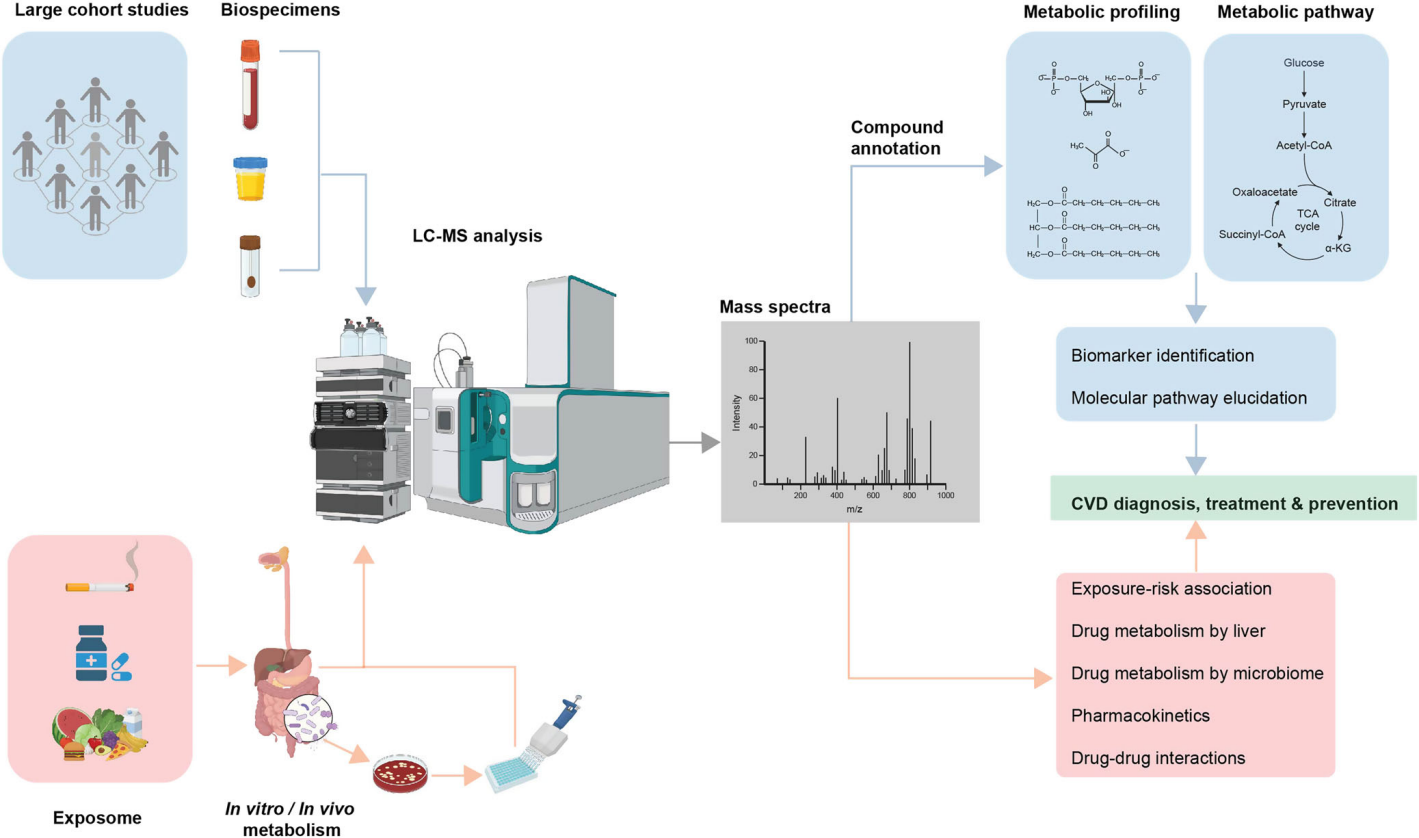
Patient-centric and intervention-centric metabolomics studies in CVD research. Patient-centric studies focus on the internal changes of the subject by characterising metabolic features of biospecimens (e.g. blood, urine, faeces) using LC-MS, the goal of which are commonly to identify clinically applicable CVD biomarkers and relevant metabolic pathways, together with other omics technologies. Intervention-centric studies focus on what a subject is exposed to that leads to the development or treatment of CVD. Depending on the nature of the intervention (e.g. environmental toxins, drugs, diet), the goal of the study can be identification of specific molecular risk factors, or tracing the metabolic fates of molecules that are beneficial or harmful for CVD prevention and treatment. Figure created with BioRender.com.

In the sections above, we discussed the motivation and application of multiomics approaches in CVD molecular mechanism and biomarker identification research nowadays. In the following sections, we focus on why metabolomics is particularly useful for studying metabolic disorders such as CVD, its history of development, and its application in biomarker identification cohort studies and other aspects of CVD research that are non-host-centric (Fig. 2).

### Metabolic disruptions in CVD

The amounts of different metabolites at any given time point can be considered reflective of the biological status of a cell/tissue/organism, and are controlled by a variety of endogenous (e.g. genetic alteration, transcriptional regulation, translational and post-translational modifications) and exogenous signals (e.g. cigarette smoke, dietary intake). Any disruptions to the healthy state as the cause or result of diseases will therefore be reflected in the levels of various metabolites. Metabolite profiling is especially valuable for CVD because most of the risk factors of CVD have their roots in disruption in the metabolic pathways and limitations with oxygen supply to the heart, which can be reflected as changes in metabolite levels in the systemic circulation. This is not surprising given that the heart is a highly active organ that converts chemical energy into mechanical energy to keep the whole body functional by metabolising various types of substrates that need to be taken up from blood circulation. Energy production in the heart is mainly via mitochondrial oxidative phosphorylation and glycolysis^84,85^ of substrates such as fatty acids (FA), lactate, glucose, ketones, and amino acids. Upon entry into mitochondria, oxidation of the different substrates convergently leads to acetyl-CoA production and entry into the tricarboxylic acid (TCA) cycle, which subsequently generates ATP via oxidative phosphorylation. A series of transporters and enzymes need to be in place to ensure the timely uptake of substrates from the bloodstream into cardiomyocytes, transport through the mitochondrial membrane and metabolic conversions inside the cells. In a healthy heart, these processes are intricately regulated by balances between intermediates (e.g. FAD/FADH2 and NAD + /NADH in the mitochondrial matrix, acetyl-CoA/CoA, ATP/AMP in cardiomyocytes) that are reflective of the current energy status in the heart. Under diseased conditions, on the other hand, dysregulation of key players in these processes leads to inefficient energy production by the heart^86^. Hence, changes in levels of the long-chain FA mitochondrial carrier, acylcarnitine, were found to be associated with risk of HF^87–91^ and other metabolic diseases such as diabetes mellitus^92–95^. Similarly, changes in blood sugar levels resulting from switching from FA metabolism to carbohydrate metabolism and ketone oxidation, and increased levels of BCAA in the plasma resulting from the inefficient oxidation also reflect disruptions in heart function^66,96–98^. Furthermore, depending on the progression of the disease, the efficiency of utilisation of different substrates will also change, which can also be captured by metabolomic profiling^99,100^ and potentially be useful for disease classification and/or risk stratification. Controlled studies comparing plasma metabolomic profiles before and after a perturbation have shed light on the impacts of myocardial ischemia and overt myocardial injury on metabolism changes^101^.

### History of metabolomics technologies

More than 95% of the modern clinical diagnostic assays are based on small molecule measurements and metabolic phenotyping had been used in diagnosis unknowingly long before the development of metabolomics technologies^102^. For example, as early as 1506, the German physician Ullrich Pinder had used the urine wheel, a catalogue of urine smell, taste and colour, essentially a qualitative summary of the urine metabolome, for disease diagnosis. Arthur Robinson and Linus Pauling first postulated the approach of quantitative analysis of the small molecules present in a person to reflect their health status in 1971^103^ with the idea that a healthy level of all the biologically essential molecules is needed to prevent disease development and that disturbances detected in the small molecule levels could guide usage of medications. The term ‘metabolic profiling’ was first used in the same year by Horning and Horning to describe urine metabolome data obtained with gas chromatography (GC) analysis^104^. In the 1980s, Nicholson and Sadler published a series of papers on the application of nuclear magnetic resonance (NMR) spectroscopy-based metabolic profiling techniques to illustrate the effects of exercise, diabetes, alcohol and other diseases on the urine and plasma metabolome of human subjects, highlighting the ease of sample preparation procedure prior to analysis and hence its ready implementation under clinical settings^105–107^. Around the same period of time, MS-based metabolic profiling had been used in the studies of bacteria and plants^108,109^. McConnell et al. applied pattern recognition in data generated from GC-MS in 1979 to differentiate urine metabolomes of diabetic individuals from healthy individuals^110^. The difficulties in detection of low- or non-volatile metabolites and the low reproducibility suffered by MS-based metabolic profiling was improved with the development of soft-ionisation methods (e.g. field desorption, laser desorption) and emission-control electronics in 1980s, which marked a step change in the field^111^. In the following decades, with advances in metabolic profiling technologies and increasing numbers of small molecules detected in body fluids, cells and tissues, the field of metabolomics emerged^112^ with the goal to augment and/or complement findings made in genomics and proteomics studies by adding the layer of dynamic metabolic status at the systemic level as discussed in previous sections. More detailed comparison between NMR spectroscopy- and MS-based technologies in metabolomics studies had been previously reviewed^113^, which mainly revolves around coverage of molecules that are present in the samples, sample preparation procedure, resolution, sensitivity, analysis time, instrument maintenance, carry-over^114,115^. In the 1980s, NMR spectroscopy first received attention in the pharmaceutical industry for toxicity testing in rodent models and around 2000, in the food industry for adulteration testing in beverages^116,117^. In diabetic cardiomyopathy and HF research, ^13^C magnetic resonance spectroscopy (MRS) has been used to noninvasively monitor metabolic flux by measuring the conversion of [1-^13^C]pyruvate into [^13^C]bicarbonate and [1-^13^C]lactate within the heart^118,119^. On the other hand, (ultra)high performance liquid chromatography (U(H)PLC) has become the powerhouse for healthcare industries and clinical studies, and will be the main focus in the following discussion.

### Metabolomics for clinical research and technical considerations

To assist more accurate clinical decision making in disease classification and medication, quantifiable biomarkers are of particular interest. In this aspect, there are two ways metabolomics technologies can be utilised. With prior knowledge of endogenous metabolic pathways, targeted quantification of selected metabolites is especially useful for hypothesis-driven research. Internal standards, which are usually isotopically labelled chemicals of the candidate metabolites, can be spiked into samples for targeted quantification of those metabolites. By tracking the quantities of metabolites over the course of disease development, risk/susceptibility biomarkers can be pinpointed; by comparing metabolite levels between control and diseased cohorts, diagnostic biomarkers can be identified; by monitoring metabolite levels over the course of a drug treatment, prognostic biomarkers can be identified.

On the other hand, without an unbiased and comprehensive capture of all the metabolites and their interactions, an accurate mechanistic understanding of disease development at the metabolic level cannot be obtained. Without such knowledge, the candidate metabolites selected for targeted metabolomics analysis might not be biologically representative and the biomarkers identified this way might be irrelevant for clinical usage. Therefore, increasing attention has been drawn to hypothesis-generating metabolomics studies, namely, untargeted metabolomics, which aims to detect as many small molecules as possible and sheds light on novel biomarkers^120^. Such studies are especially facilitated by the technological advancements that have improved mass spectrometers’ sensitivity, mass accuracy, resolution, and acquisition speed.

To achieve optimal coverage of metabolites in various biological samples with untargeted metabolomics on LC-MS platforms, several technical considerations need to be taken into account. Prior to LC-MS analysis, metabolites first need to be extracted from the samples. Ideally, this procedure maximises the solubility of all metabolites while excluding larger molecules such as proteins and lipids. Samples are then subjected to LC-MS analysis, where a liquid chromatography system is directly coupled to the MS. During the chromatographic process metabolites are separated, depending on their individual physicochemical properties, the dimensions and the stationary phase of the column and the mobile phases used. For example, hydrophilic interaction chromatography (HILIC) is suited for the analysis of polar molecules, whereas reverse-phase (RP) chromatography is suited for nonpolar molecules. Despite being time-consuming for high-throughput studies, liquid chromatography helps sensitive detection of individual molecules by MS, and the individual elution times of the compounds from the column (retention time) provide an additional piece of information for confident downstream compound annotation. Alternatively, at the expense of sensitivity, samples can be directly injected into the MS without LC separation (known as flow-injection mass spectrometry (FI-MS)) to improve the analytical throughput^121^. This, however, comes at the cost of inability to separate isobaric compounds.

The assignment of metabolite identities from their MS signals is based on their mass-to-charge ratios acquired with MS1 as well as their fragmentation patterns acquired with MS2. Currently, two acquisition methods providing good coverage of the MS1 and MS2 space have received great attention - data-dependent acquisition (DDA) and data-independent acquisition (DIA). The pros and cons of both acquisition modes have been extensively reviewed elsewhere^122,123^. Briefly, DIA allows fragmentation of all MS1 ions and hence provides fragmentation spectra of all ions at their respective retention time, providing maximal coverage of ions present in the MS2 space. However, associating precursor ions with their fragmentation patterns has been proven challenging in practice. DDA, on the other hand, partially circumvents this problem by selecting MS1 precursor ions for fragmentation with narrow isolation windows based on preset criteria such as intensity, resulting in better data quality of the MS2 spectra and subsequently improving the confidence in molecular identification and annotation. The downside of DDA, however, is that depending on parameter settings, peak detection algorithms and compound intensity in a given sample, it can be prone to undersampling, resulting in missing values and limited reproducibility.

Assuming the best acquisition of both MS1 and MS2 spectra with high sensitivity and resolution, annotation of the detected molecules still presents a bottleneck for meaningful biological interpretation, as a large proportion of metabolites uncovered by untargeted metabolomics are not easy to annotate and/or are only ambiguously annotated. As reviewed by Cai et al.^124^, metabolite annotation typically relies on comparing acquired spectra with spectral libraries obtained from chemical standards (e.g. METLIN), which are often not comprehensive and poorly curated. To bridge the gap in identification of unknown biological molecules, *in-silico* prediction of MS2 spectra and/or molecular fingerprints (e.g. CFM-ID, SIRIUS)^125,126^ and network-based approaches based on known metabolic reaction networks^127^ have become more mature in recent years. A practical strategy to increase the quality and efficiency of annotation in large cohort samples is to perform careful compound annotation on pooled QC samples first using both MS1 and MS2 spectra, to obtain a well-curated metabolite annotation list, which is subsequently used to annotate signals from large cohort samples based on MS1 spectra only by matching the mass and retention time of individual molecules^128^.

Aside from the technical considerations in untargeted metabolomics analysis, great care should be taken to ensure comparability between datasets and high fidelity in the conclusions drawn. These can be affected, amongst other factors, by batch effects and the lack of available internal standards for some of the metabolites (and hence absolute quantification). This is especially important for biomarker discovery in population-based epidemiology studies which usually contain large sample sizes to boost the statistical power or have samples obtained at longitudinal timepoints. Consequently, the analysis of samples often needs to be divided into smaller batches. In such cases, batch correction strategies (e.g. quality control (QC) samples, replicate samples) are required to ensure that signals can be compared across different batches. Additionally, as for other omics technologies, the statistical models need to be scrutinised for correct incorporation of all the confounding factors to ensure the validity of biomarkers identified. We encourage the readers to refer to previous literature reviews for detailed explanations of these post-experimental analysis procedures^129,130^.

In practice, a combination of hypothesis-generating untargeted metabolomics and hypothesis-driven targeted metabolomics approaches using animal models and human cohort samples is commonly deployed for novel biomarker and pathway identification^131^. For example, the discovery of trimethlyamine-*N-*oxide (TMAO) as a target for atherosclerosis followed the finding of higher TMAO levels in more advanced atherosclerotic plaques in a genetically deficient atheroprone mouse model^132^. This was confirmed in a subsequent study in a cohort of age- and gender-matched patients and controls using untargeted metabolomics, where a panel of metabolites were identified to be associated with CVD risks, among which is TMAO^133^. Two other dietary phosphatidylcholine metabolites, betaine and choline, were found to be correlated with TMAO, suggesting the involvement of a specific biochemical pathway. This was subsequently confirmed in an interventional study where healthy human participants were given isotope-labelled dietary phosphatidylcholine with or without oral broad-spectrum antibiotics. Tracing the isotope-labelled metabolites of phosphatidylcholine with targeted LC-MS then elucidated the involvement of gut microbial metabolism in regulating TMAO circulation levels in plasma and in urine^134^. Another example is the recent study uncovering the link between terminal breakdown metabolites of excess niacin with CVD risk, where the authors first identified the candidate metabolites and their associated pathways in a prospective cohort to be associated with CVD risk with untargeted metabolomics and network module-based pathway analysis, then they confirmed the association with two distinct validation cohorts with a stable-isotope-dilution LC–MS/MS method^135^.

The predictive value of biomarkers identified with metabolomics approaches for specific subtypes of CVDs up until 2020 has been systematically reviewed elsewhere, which we encourage the readers with an interest to read further^136–138^. Some recurring metabolites that are associated with the risk of multiple CVD conditions (e.g. myocardial infarction, congestive heart failure, CAD, stroke, transient ischemic attack, peripheral artery disease, unstable angina) include acylcarnitines, BCAA, glutamate and ceramides, etc. In the remainder of this paragraph we will elaborate on some of the more recent studies of specific CVD conditions, where LC-MS-based metabolomics had been the workhorse for novel predictive or diagnostic biomarkers identification. In an atherosclerosis study performed by Sardar et al. using LC-MS, taurocholic acid (TCA), cholic acid, cortisol, hypoxanthine, TMAO, and isoleucine were observed to be upregulated in serum of atherosclerosis patients, while glycoursodeoxycholic acid, glycocholic acid, testosterone, leucine, methionine, phenylalanine, tyrosine, and valine were downregulated^139^. Similarly, Su et al. used serum metabolomics to assess carotid intima-media thickness (C-IMT), a proxy of subclinical atherosclerosis, in a cohort of 462 T2DM patients. The authors found higher levels of deoxycholic acid (DCA) and taurodeoxycholic acid (TDCA) and lower levels of TCA in patients with abnormal C-IMT, proposing the use of serum bile acid levels as potential biomarkers^140^. Gamma-glutamyl-glutamic acid and homovanillic acid sulfate were shown to associate with atherogenic risk state in a study with 302 adults analysed by FI-MS-based metabolomics^141^, while a study by Ottosson et al. on 6865 individuals from two Swedish population based cohorts puts acylcarnitines, dimethylguanidino valerate (DMGV), glutamate and cysteine forward as predictors for aortic stiffness^142^. DMGV has also been seen to be strongly associated with the presence of calcified plaque in a study performed by Vernon et al. investigating CAD in plasma of 1002 patients by LC-MS-based metabolomics. They additionally reported positive associations between glutamate levels and non-calcified plaques, phenylalanine levels and amount of CAD and a negative association between TMAO and non-calcified plaque^143^. Altered levels of amino acids were also observed in a study by Wang et al., using LC-MS to characterize serum samples from patients with acute ischemic stroke (AIS) in a relatively small cohort of 49 patients with large artery atherosclerosis, 50 patients with small artery occlusions and 50 healthy controls. Their data suggested glutamine, arginine and proline as well as oleic acid, linoleic acid, arachidonic acid to be potential biomarkers^144^. Interestingly, arachidonic acid and its metabolite leukotriene B4 has also been observed to be elevated upon development of atherosclerosis in a study of 38 atherosclerosis patients matched with 35 healthy controls in a study by Ma et al.^145^. Using a mouse model they observed a reduced plaque stability upon treatment with an arachidonic acid inhibitor, aspirin. This led the authors to speculate that arachidonic acid and leukotriene B4 may promote migration and adhesion of white blood cells to the arterial wall. Lee et al. demonstrate the discriminatory power of the amino acids lysine, serine and threonine in subtyping stroke in a targeted LC-MS-based study of 346 participants, specifically aiming to differentiate between large-artery atherosclerosis (LAA, *N* = 169) and cardioembolic stroke (CE, *N* = 147), in comparison to healthy adults (*N* = 30). Other discriminatory compounds found were kynurenine, putrescine and lysophosphatidylcholine acyl C16:0^146^. Various studies showed the involvement of lipids and fatty acids in ischemic stroke^147^, myocardial infarction^148,149^ and asymptomatic intracranial arterial stenosis^150^. Zagura et al. used targeted, quantitative metabolomics to profile metabolic alteration in elderly patients with peripheral arterial disease (PAD), which was the first study that found metabolic signatures that could differentiate patients at advanced disease stages from healthy controls^151^. Lactate, free carnitine and some amino acids including tyrosine were higher in PAD patients, while pyruvate, citrate, α-ketoglutarate, aconitate and cysteine were lower. Targeted, quantitative metabolomics was also used in investigating the metabolic determinants of atrial fibrillation (AF) using samples from 5688 study participants, including 64 AF cases in the Cooperative Health Research in South Tyrol study. Among the 175 metabolites measured, lysoPC a C20:3 was the only one that showed a significant difference between cases and controls (41% reduction with adjusted *p*-value: 0.005)^152^. A similar, yet substantially smaller prospective cohort study (*N* = 54) found arachidonic acid, glycolic acid, serine and palmitelaidic acid to be potential biomarkers for AF and its subtypes^153^. Disrupted metabolism of serine has also been shown to be associated with ventricular arrhythmia by Yang et al. together with glycine, threonine and BCAAs^154^. Given the link between macrophage infiltration and aortic aneurysm and dissection (AAD) development and the role of succinate in triggering inflammatory changes in macrophages, Cui et al. first used untargeted metabolomics to show that succinate was the most upregulated metabolite in AAD patients. The authors further investigated the mechanism underlying succinate accumulation in macrophages using mouse models and proposed a inhibition pathway in macrophages to prevent succinate accumulation in AAD tissues, serving as a potential therapeutic target^155^.

In addition to establishing high-throughput workflows in individual labs for CVD biomarker identification, cross-institutional and cross-continental collaborative meta-analyses presents added value in increasing the confidence of the potential biomarkers identified independently. One such study focussing on metabolites for incident myocardial infarction (MI)^156^, for example, combined blood plasma data obtained from six intercontinental cohorts. Different platforms and technologies (e.g. NMR vs LC-MS, GC-MS vs LC-MS, targeted vs untargeted) were used in each of the studies. Correlational analyses between circulating metabolites and incident MI for each cohort were performed independently and results could then be pulled together. Metabolite hits identified in the multiple-testing adjusted pathway enrichment analyses were then compared with previous studies, where the robustness of the meta-analysis could be assessed. The study confirmed the negative effect of the BCAA isoleucine on MI and the protective effects of nonessential amino acids such as glutamine, glycine, and serine. Novel metabolites belonging to lipid, xenobiotic and nucleotide classes were also uncovered in this study.

The added value of metabolic profiling on risk stratification has also been assessed in a study that derived the states of 168 circulating metabolic markers in 117,981 participants with 24 common conditions including, coronary heart disease (CHD), cerebral stroke, HF, AF and PAD using machine learning and evaluated the improvement on outcome prediction with or without the metabolic contribution alongside the established predictors such as age, sex, the American Heart Association (ASCVD) predictors, etc^157^. The authors have shown that despite the fact that the established biomarkers tend to have comparable or better discriminative power than metabolic states alone for most of the CVD included, the combination of metabolic states and traditional predictors tend to stably improve the discriminative performance across different age groups and sexes.

With the examples discussed above, we showcase the value of metabolomics technologies in not only clinical research for biomarker validation and identification but also in clinical settings where some of the existing knowledge obtained with metabolomics can be readily implemented in CVD disease classification and risk stratification.

### Metabolomics in CVD research other than biomarker identification

So far we focussed on the utilisation of multiomics technologies in measuring endogenous metabolites, where the goal is mainly to capture changes at different molecular levels in the body in response to interventions and then use such information to derive useful clinical biomarkers, which in turn guide further clinical actions. These interventions can encompass the occurrence of a disease itself, the various risk factors that lead to disease development (e.g. genetic mutations, intake of dietary compounds, exposure to environmental chemicals such as cigarette smoke) or the treatment for the disease such as medications. Unlike other omics technologies (genomics, transcriptomics and proteomics), metabolomics can not only characterise molecular alterations within the system in response to interventions but also some of the interventions themselves. This intervention-centric approach can bring additional value for prevention and treatment of CVD by monitoring exposure to xenobiotics and their biotransformation and interactions within the patient.

Exposome studies focus on comprehensive characterisation of human environmental exposures^158^, which include but are not limited to dietary intake and smoking, two of the major risk factors for CVD development discussed in the previous section. Many studies have associated specific chemical groups with CVD risk, but correlation between dietary patterns or environmental exposure patterns and CVD risk has not yet been studied to great extent at the molecular level^159^. However, given that we are constantly exposed to mixtures of chemicals, comprehensive characterisation of such exposure patterns at the molecular level in tissues such as blood and urine with targeted and untargeted metabolomics can help to more accurately associate how specific exposures relate to risk. Additionally, other than directly measuring xenobiotics in biological samples such as plasma and urine, measuring the adduct they form with endogenous macromolecules such as albumin can more precisely reflect chronic exposures. This approach, known as adductomics, has been proven useful for monitoring albumin adducts formed due to air pollution^160,161^, which can potentially be incorporated in correlational studies for CVD risk assessment and biomarker identification.

Other than correlational studies focussing on exposure to dietary compounds and environmental toxins, metabolomics tools are particularly useful for monitoring the biotransformation and kinetics of xenobiotics within the host system. In the context of drug development, the ADME (absorption, distribution, metabolism and excretion) processes of drug candidates and their biotransformed products can be absolutely or relatively quantified with metabolomics and mechanistically linked to functional readouts such as efficacy and toxicity^113^, which in turn facilitates future *in-silico* drug candidate screening before any experimental testing. Such knowledge then has the potential to reduce the rate of drug failure in clinical trials.

Traditionally, most of the biotransformation studies have focussed on the liver as the key metabolic hub in the human body. Most of the preclinical studies deploy hepatic models such as microsome, primary human hepatocytes and 3D cell culture, to determine the primary metabolic conversion products of drug candidates^162^. More recently, an emerging field, the study of the gut microbiome, has shed light on the metabolism of clinical drugs by the ‘extra organ’, gut microbiota, which colonises our gastrointestinal tract^163^. The human gut microbiome consists of roughly 1000 bacterial species, encodes 100 times more genes than the host^164^ and possesses a remarkable metabolic potential that will significantly contribute to host metabolism of xenobiotics. To probe the species-specific metabolic activities in such highly diverse microbial communities, high-throughput metabolomics techniques present a great advantage. Using high-throughput targeted UPLC-MS analysis, a study has found more than two-thirds of 271 clinically used and chemically diverse drugs to be metabolised by at least one of 76 tested human gut bacterial strains^165^. The cholesterol-lowering drug lovastatin used in CVD treatment was one of the positive controls, which was metabolised by most of the tested strains^165^. To then pinpoint the bacterial gene products (or pathways) responsible for the metabolic transformation of the CVD drug, targeted screening can be applied. Depending on whether such bacterial-mediated alterations in drug pharmacokinetics is favourable or harmful for the treatment outcome, the respective bacterial genes can be used as biomarkers when tailoring treatment regimes for individual patients. In addition to the metabolic potential encoded in the microbiome itself, its interactions with host metabolism is also an important aspect of pharmacokinetic studies as the presence of enterohepatic circulation means that the metabolic products from host metabolism can be substrates for microbial metabolism and vice versa^166^. Such host-microbe interactions will affect the bioavailability of drugs and hence treatment outcomes. By quantifying metabolite levels in the microbiota and in various host organs, the contribution of host and microbial metabolism of a given drug can potentially be disentangled for more accurate pharmacokinetic modelling^167^.

As mentioned above, humans are simultaneously exposed to a mixture of xenobiotics, and therefore, other than probing the metabolic fates of drugs and other xenobiotics independently upon their entry into the host, it is also important to understand how the interactions between various xenobiotics can affect the metabolism and functionality of each of them. Drug-drug interactions (DDI), for example, have received great attention in CVD clinical research due to adverse effects that have been observed in patients co-administering different drugs. The combined usage of antihypertensives such as angiotensin-converting enzyme inhibitors (ACEI), angiotensin receptor blockers and diuretics, for instance, can lead to acute kidney injury^168,169^. Metabolic profiling in controlled in vivo studies can provide valuable insights into the molecular mechanisms underlying these observations. For instance, it was found that metabolic activation via hydroxylation of the above-mentioned CVD drug lovastatin was downregulated upon antibiotic-treatment in rats^170^, which would suggest that the systemic exposure of the activated form of lovastatin will be lower in patients who are undergoing long-term antibiotic treatments. Large human cohort studies comparing drug metabolite levels and treatment outcomes in patients taking lovastatin with or without antibiotic co-treatment can be performed based on hypotheses generated from such animal studies to further validate the translational value of such DDI. If translatable, knowledge derived from such studies can guide the usage and dosage of CVD drugs in patients taking other medications already and thus avoid adverse effects and maximise treatment efficacy on an individual basis.

## Concluding remarks and future perspectives

Mechanistic understanding of diseases at the molecular level is essential in order to achieve disease prevention, classification and treatment prognosis on a personalised basis. For complex diseases with multifactorial causes such as CVD, the integration of information from different biological layers, ranging from genetic polymorphism to temporal metabolic alterations in large sample sets, is of particular value, to identify the statistically significant and biologically relevant pathways and to capture interindividual variations within different biological processes. On the other hand, metabolomics on its own is a powerful tool for various aspects of CVD research, especially because the majority of the CVD risk factors have their roots in metabolic disruptions. Furthermore, metabolism of both naturally occurring molecules (e.g. FA, glucose, amino acids) and xenobiotics (e.g. drugs, toxins) can all affect host physiology and metabolic phenotypes. Overall, metabolomics opens up a window of opportunity to gain insights into both individual metabolic pathways and their interactions with one another for a more comprehensive pharmacokinetic and functional interpretation, by monitoring the metabolic fates of various types of small molecules in different body compartments. This is not only beneficial for the patients, but also for pharmaceutical companies to identify new drug targets and to select for drug candidates with lower failure rates in clinical trials.
